# Alterations in Brain Connectivity Underlying Beta Oscillations in Parkinsonism

**DOI:** 10.1371/journal.pcbi.1002124

**Published:** 2011-08-11

**Authors:** Rosalyn J. Moran, Nicolas Mallet, Vladimir Litvak, Raymond J. Dolan, Peter J. Magill, Karl J. Friston, Peter Brown

**Affiliations:** 1 Wellcome Trust Centre for Neuroimaging, Institute of Neurology, University College London, London, United Kingdom; 2 Medical Research Council Anatomical Neuropharmacology Unit and Oxford Parkinson's Disease Centre, University of Oxford, Oxford, United Kingdom; 3 Department of Clinical Neurology, University of Oxford, Oxford, United Kingdom; Northwestern University, United States of America

## Abstract

Cortico-basal ganglia-thalamocortical circuits are severely disrupted by the dopamine depletion of Parkinson's disease (PD), leading to pathologically exaggerated beta oscillations. Abnormal rhythms, found in several circuit nodes are correlated with movement impairments but their neural basis remains unclear. Here, we used dynamic causal modelling (DCM) and the 6-hydroxydopamine-lesioned rat model of PD to examine the effective connectivity underlying these spectral abnormalities. We acquired auto-spectral and cross-spectral measures of beta oscillations (10–35 Hz) from local field potential recordings made simultaneously in the frontal cortex, striatum, external globus pallidus (GPe) and subthalamic nucleus (STN), and used these data to optimise neurobiologically plausible models. Chronic dopamine depletion reorganised the cortico-basal ganglia-thalamocortical circuit, with increased effective connectivity in the pathway from cortex to STN and decreased connectivity from STN to GPe. Moreover, a contribution analysis of the Parkinsonian circuit distinguished between pathogenic and compensatory processes and revealed how effective connectivity along the indirect pathway acquired a strategic importance that underpins beta oscillations. In modelling excessive beta synchrony in PD, these findings provide a novel perspective on how altered connectivity in basal ganglia-thalamocortical circuits reflects a balance between pathogenesis and compensation, and predicts potential new therapeutic targets to overcome dysfunctional oscillations.

## Author Summary

Parkinson's disease is a progressive age-related neurodegenerative disorder that severely disrupts movement. The major pathology in Parkinson's disease is the degeneration of a group of neurons that contain a chemical known as dopamine. Treatment of Parkinsonism includes pharmacological interventions that aim to replace dopamine and more recently, implanted devices that aim to restore movement through electrical stimulation of the brain's movement circuits. Understanding the electrical properties that emerge as a result of depleted dopamine may reveal new avenues for developing these technologies. By combining a novel model-based approach with multi-site electrophysiological recordings from an animal model of Parkinson's disease we provide empirical evidence for a link between abnormal electrical activity in the Parkinsonian brain and its physiological basis. We have examined the connections along the brain's motor circuits, and found an abnormality in inter-area connections in a particular neural pathway, a pathway critically dependent on dopamine. The scheme makes strong and testable predictions about which neural pathways are significantly altered in the pathological state and so represent empirically motivated therapeutic targets.

## Introduction

In Parkinson's disease (PD), degeneration of midbrain dopamine neurons severely disrupts neuronal activity in looping circuits formed by cortico-basal ganglia (BG)-thalamocortical connections [1,2,3]. Studies have shown that excessive oscillations at beta frequencies (13–30 Hz) are a key pathophysiological feature of these Parkinsonian circuits, when recorded at the level of unit activity and/or local field potentials (LFPs) in several key circuit nodes. These nodes include the frontal cortex, subthalamic nucleus (STN), external globus pallidus (GPe) and internal globus pallidus (GPi) [4,5,6,7,8,9]. Suppression of pathological beta-activity is achieved by dopamine replacement therapies [10] and surgical treatments e.g. high-frequency, deep brain stimulation (DBS) of the STN; where prolonged attenuation after stimulation is observed [11,12]. Bradykinesia and rigidity are the primary motor impairments associated with beta activity and, following dopamine replacement therapies, improvements in these motor deficits correlate with reductions in beta power [13,14,15,16]. Moreover, a recent report has shown that stimulating the STN at beta frequencies exacerbates motor impairments in Parkinsonian rodents [17], in line with similar findings in PD patients [18,19].

Precisely how dopamine depletion leads to abnormal beta power is unknown. Recent work in rodents has revealed that excessive beta-activity emerges in cortex and STN after chronic dopamine loss but not after acute dopamine receptor blockade [5,8]. Here, we examine whether changes in effective connectivity between the nodes of the cortico-basal ganglia-thalamocortical network can account for enhanced beta oscillations following chronic dopamine loss. To test this hypothesis we used dynamic causal modelling (DCM). This approach allows one to characterise the distributed neuronal architectures underlying spectral activity in LFPs. DCM is a framework for fitting differential equations to brain imaging data and making inferences about parameters and models using a Bayesian approach. A range of differential equation models have been developed for various imaging modalities and output data features. The current library of DCMs includes DCM for fMRI, DCM for event related potentials and DCM for steady state responses (DCM-SSR). The current paper is based on DCM-SSR, designed to fit spectral data features [20,21].

Using spectral data, recorded simultaneously from multiple basal ganglia nuclei and the somatic sensory-motor cortex, we asked whether systematic changes in re-entrant neural circuits produce the excessive beta oscillations observed in LFPs recorded from the 6-hydroxydopamine (6-OHDA)-lesioned rat model of PD [2,5,22]. We inverted the models (i.e., optimised the model parameters or “fit” the data) using LFP data collected simultaneously from electrodes implanted in frontal cortex, striatum, GPe and STN. Specifically, we used neural mass models that characterise the main projection cell types at each circuit node as glutamatergic or GABAergic. Neural mass models describe neuronal dynamics in terms of the average neurophysiological states (e.g., depolarisation) over populations of neurons. Inference on effective connectivity differences observed between the Parkinsonian and control cases was based on *a posteriori* estimates of connectivity and synaptic parameters (i.e., the most likely given the data). Using these estimates, we characterised the sensitivity of beta oscillations to changes in particular connection strengths to identify candidate connections that may represent therapeutic targets in idiopathic PD.

Measures of functional connectivity have been applied previously to examine frequency-specific signal correlations between nodes in the cortico-basal ganglia-thalamocortical network. These measures have highlighted excessive coupling between the cortex and STN [22] and between STN and GPe [5,22] in animal models of PD. While functional connectivity and effective connectivity measures share some technical aspects e.g. likelihood models [23] or Bayesian estimators [24], the underlying concepts are fundamentally different [25]. The distinction between functional connectivity (a descriptive characterisation of the statistical dependence between two time series) and effective connectivity (a model-based characterisation of causal influences) emerged from the analysis of electrophysiological time series: Aertsen et al. [26], used the term effective connectivity to define the neuronal interactions that could explain observed spike trains using a “minimum simple neuronal model”. In what follows, we employ such a minimum model approach, using the key elements of known cortico-basal-ganglia-thalamocortical interactions. Our model predicts the output of this loop circuit *in vivo*, where we assume observed responses are caused by interactions among neuronal populations or sources, with known neurotransmitters and directed connections. The starting point for analyses of effective connectivity in this paper is the end point of classical functional connectivity analyses; namely, observed cross-spectral densities (and their associated cross-correlation and coherence functions). In other words, we place special emphasis on explaining how functional connectivity emerges in terms of directed connections that rest on a particular model of neuronal interactions. In what follows, we illustrate this approach when applied to the directed circuitry of a cortico-basal ganglia-thalamocortical system.

## Results

### Dynamic Causal Modelling of the Cortico-Basal Ganglia-Thalamocortical Loop

**Figure 1:**
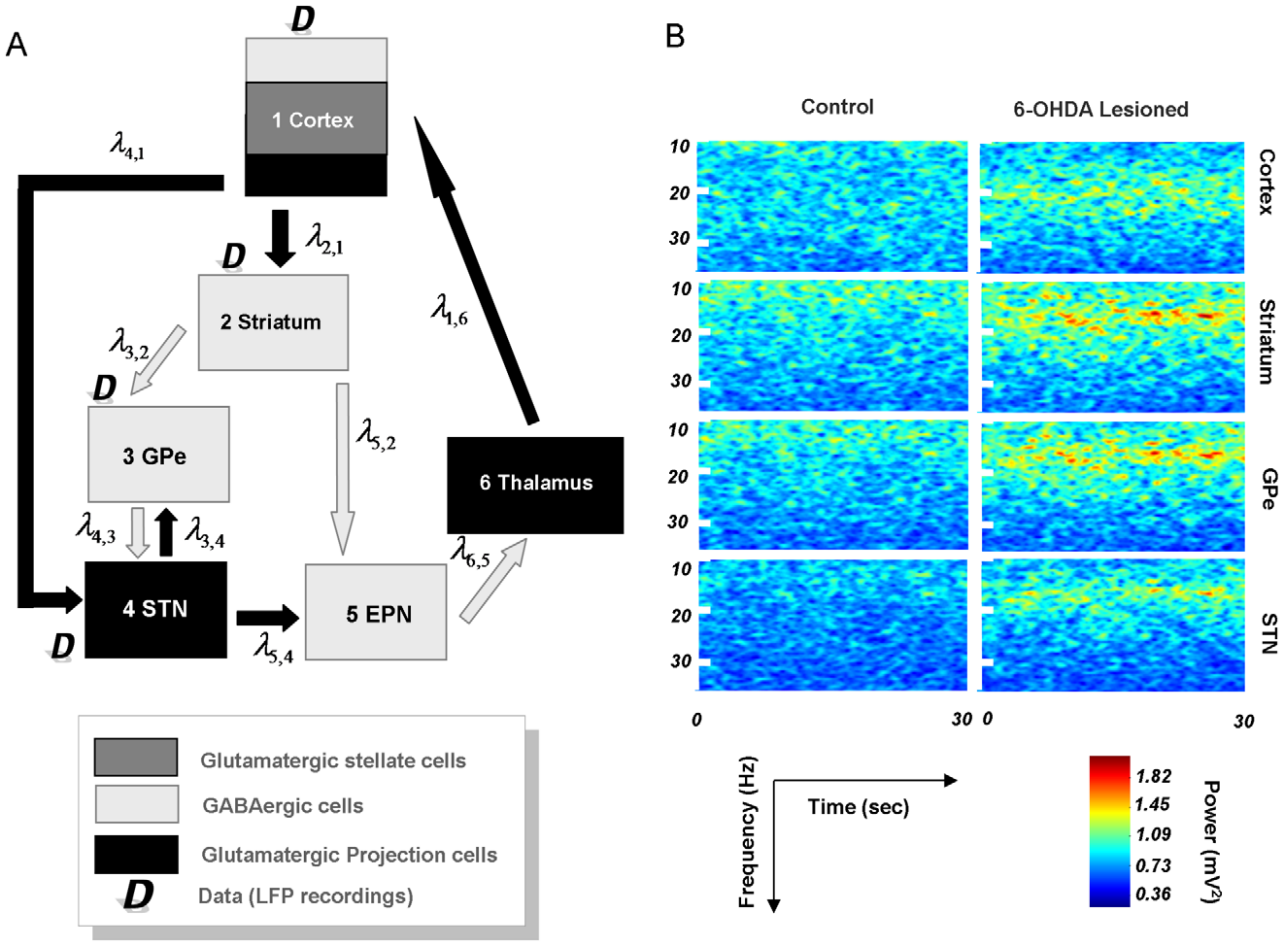
Structure of dynamic causal model and spectral data. (A) The structure of the DCM encompasses the principal nodes and connections in the rodent cortico-basal ganglia-thalamocortical loop. The nodes include a cortical source, *1*, modelled by a three subpopulations corresponding to excitatory input (glutamatergic spiny stellate) cells, projection (glutamatergic pyramidal) cells and inhibitory (GABAergic) interneurons. Excitatory projections from cortex innervate the Striatum *2*, and STN, *4* (the hyperdirect pathway). The Striatum comprises an inhibitory subpopulation that projects to two other inhibitory sources, GPe *3* (as part of the indirect pathway), and EPN *5* (via the direct pathway) with the latter being a BG output nucleus. The GPe and STN express reciprocal connections, and signals from the hyperdirect and indirect pathways are transformed to BG output nuclei via excitatory STN projections to the EPN. The thalamus, *6*, which excites cortex, is itself inhibited by connections from EPN. Data, *D*, used for the model inversion were acquired from LFP recordings in cortex, Striatum, GPe and STN. (B) Group average spectrogram of LFP data recorded from cortex, striatum, pallidum and subthalamic nucleus. *Left* Control animals display low power broadband signals across all thirty seconds. *Right* Parkinsonian animals display high amplitude beta power in all the electrode recordings that extends throughout the epoch.

**Figure 2:**
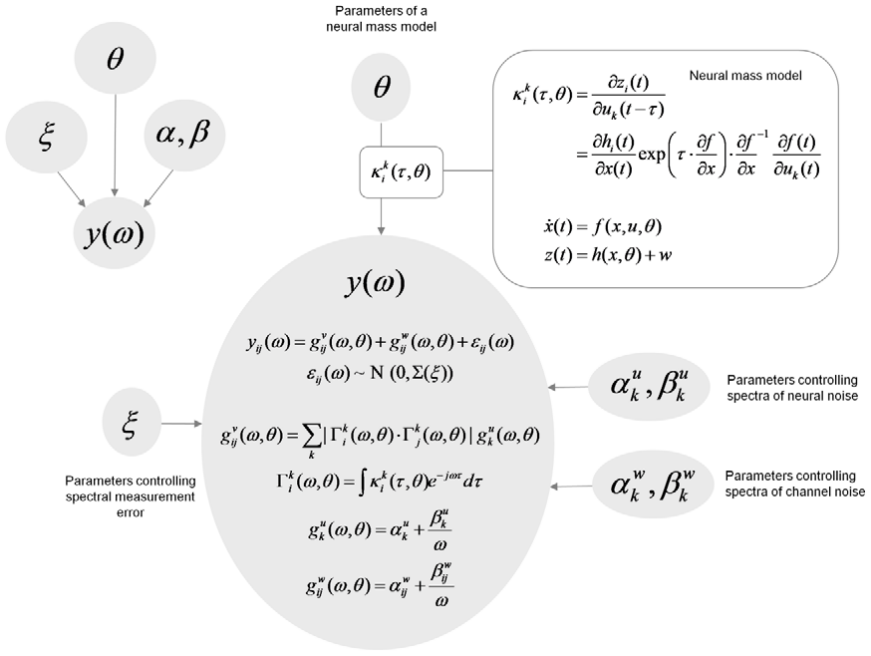
Generative model. This figure summarises the generative model as a Bayesian Network (upper left insert), which has been unpacked to show the form of the conditional dependencies in terms of the equations of the generative model (lower right). This model provides a probabilistic description of data in terms of the parameters that cause them. These include parameters controlling the spectral density of neuronal and channel noise and parameters controlling the variance of observation error on the cross-spectra predicted. The parameters of interest pertain to a neuronal model that is cast as a continuous-time state-space model. It is this neuronal model that constrains the mapping from neuronal noise or innovations to observed cross-spectra. The details of the neuronal (mass) model are provided in the next figure. Please see the main text for a detailed explanation of the equations that define the conditional dependencies.

**Figure 3:**
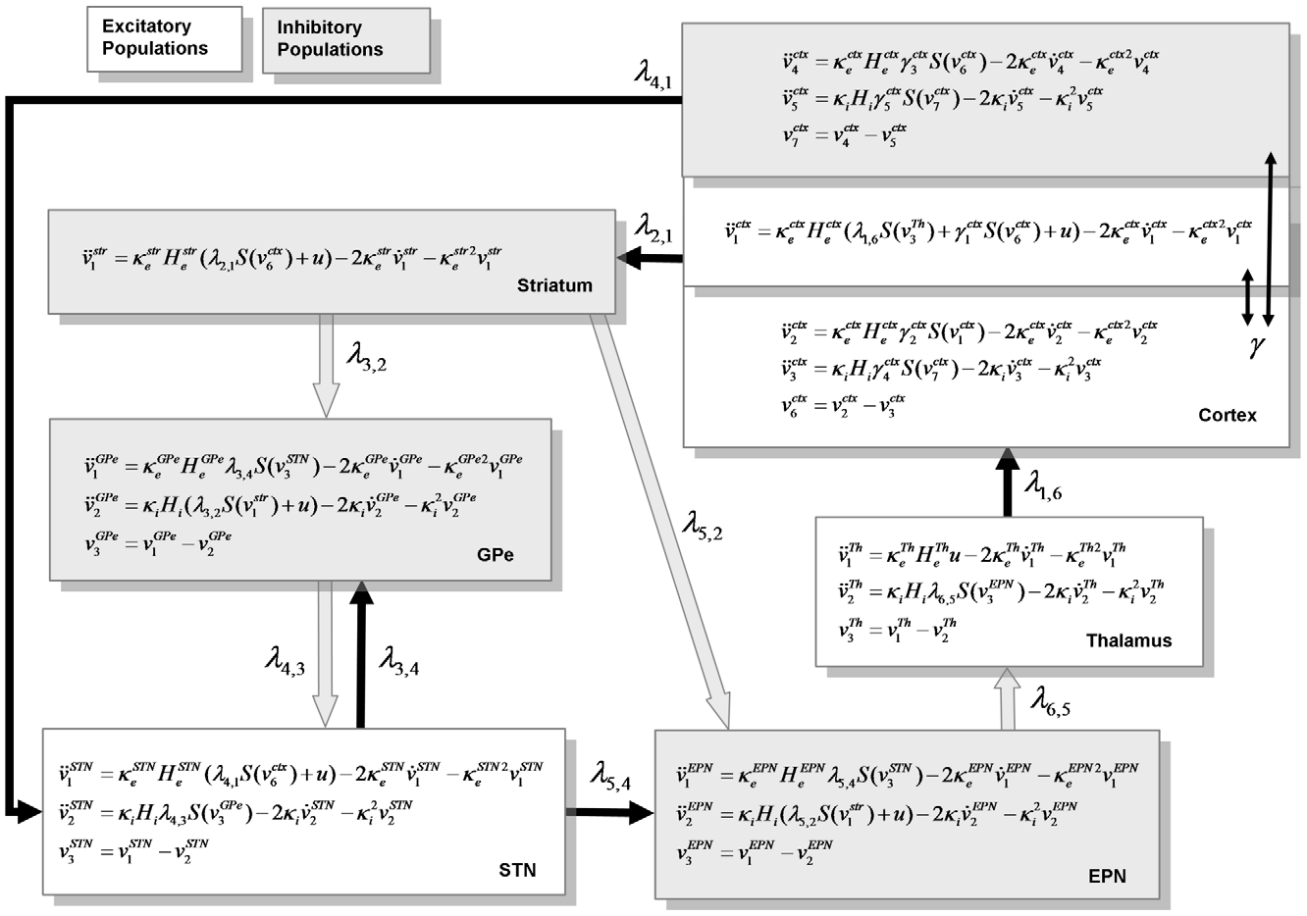
Neural mass model of cortico-basal ganglia-thalamocortical circuit. These are the differential equations modelling the hidden neuronal states in the subpopulations comprising the nodes of the circuit model. These equations take the form of Equation 7. For simplicity, we have dropped the dependency on time and have therefore omitted the delays (see Equation 7). In the cortex, the parameters 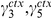 describe the intrinsic connections from the pyramidal cells within the same cortical source and reciprocal inhibitory afferents respectively and 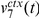 represents the average membrane potential of inhibitory cells. In the granular layer, spiny stellate cells receive excitatory connections from the thalamus, with strength 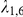 and excitatory inputs from pyramidal cells within the same region 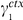. In turn, pyramidal cells in the cortex receive inhibitory inputs from the interneurons within that cortical region and excitatory inputs from the stellate cells within that region mediated by intrinsic connectivity parameters 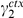 and 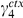 respectively. The pyramidal cells from the cortex send efferent extrinsic connections to the basal-ganglia, arriving at the striatum with a strength parameterised by 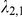 and to STN, with strength 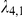. At the striatum, one inhibitory subpopulation models GABAergic projection neurons with input from cortex. These striatal GABAergic projection neurons then inhibit both GPe and EPN, such that the dynamics in the GPe are modelled as an inhibitory network with input 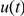 and connections from the STN with effective connectivity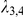. This population is inhibited by the striatum with an effective connectivity of 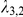. The STN is reciprocally connected to GPe, receiving inhibitory connections mediated by 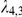 and is excited by the cortex via the hyperdirect pathway with effective connectivity 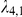. The EPN receives inputs from both STN (glutamatergic) 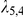 and the striatum (GABAergic) 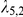
and sends feedforward inhibition to the thalamus 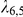. Excitatory connections from thalamus to cortex complete the closed loop architecture.

Dynamic causal models for LFP data typically comprise connected cortical sources, where each source is described by a neural mass [27]. This neural mass ascribes point estimators to hidden neuronal states (ensemble depolarisation and firing rates), capturing the average activity of a population of neurons [28], i.e. a mean-field approximation. These dynamics depend on model parameters that encode, for example, inter-regional connectivity, the amplitude of postsynaptic responses and/or synaptic rate constants. Here, three (layered) populations were used to model the cortical source, while a single population of neurons, (either glutamatergic [excitatory] or GABAergic [inhibitory]) was used for distinct BG nuclei (see Figure 1A). The differential equations describing neuronal dynamics in the basal ganglia and cortex are identical in their form but have different parameters (see Figures 2 and 3). These equations model the postsynaptic convolution of presynaptic inputs by an implicit postsynaptic kernel. The ensemble firing of one population changes the average membrane potential of another, depending on whether it uses glutamate or GABA as a neurotransmitter. Glutamatergic inputs are assumed to produce postsynaptic depolarisation, while GABAergic inputs are assumed to be hyperpolarising. These effects are mediated by a postsynaptic (alpha) kernel that is either positive or negative, respectively. This (excitatory or inhibitory) influence of one population on another is parameterised by extrinsic connectivity (between distinct nodes; e.g. from cortex to STN) or intrinsic connectivity (between different subpopulations within a node; e.g., cortex). See Materials and Methods. In short, effective connectivity is modelled as a gain factor that couples discharge rates in one population to membrane potentials in another.

The connections in our standard DCM were based on the well characterised re-entrant circuits linking the cortex, basal ganglia and thalamus in rodents and primates (Figure 1A). The main features of this network include the so-called ‘direct’, ‘indirect’ and ‘hyperdirect’ pathways [29,30]. The striatum, the primary input station of the BG, receives glutamatergic afferents from cortex and is composed primarily of GABAergic projection neurons. The striatum transmits cortically-derived information to the BG output nuclei; the entopeduncular nucleus (EPN) (homologous to the internal globus pallidus in primates), and substantia nigra pars reticulata, via the polysynaptic indirect pathway and the monosynaptic direct pathway (Figure 1A). In the former, striatal neurons innervate GABAergic GPe neurons which, in turn, innervate glutamatergic STN neurons (that then project to the BG output nuclei). Although not considered in the classic feed-forward organisation of the direct-indirect pathway schemes, we include here the feed-back projection from STN to GPe, because these two nuclei are more realistically embodied in a reciprocally-connected network [31], particularly in the context of excessive beta oscillations [6]. These three structures along the indirect pathway were modelled by ensembles of inhibitory neurons (in the striatum and GPe) and a population of excitatory neurons (in the STN). Though neurons in striatum and GPe are functionally distinct [7], we allow the data to dictate any differences in their synaptic properties. In the direct pathway, the striatum directly inhibits GABAergic EPN neurons, which also receives excitatory input from STN. While no data were acquired from the EPN, we included an inhibitory population connected to an excitatory thalamic mass to complete the closed loop dynamics [29]. These two nodes were modelled as ‘hidden sources’ because inferences can still be made about the parameters of hidden sources, based on the influence they exert on nodes from which LFP recordings are made. It is important to note that mathematically, all the parameters of a DCM are hidden or latent (i.e. cannot be accessed directly from the data, [32]) and the full dataset serves to optimise all of the parameters of the model. In other words, while LFP recordings (representing noisy dynamical state measurements) from the EPN and thalamus would further constrain and improve parameter optimisation, we can still infer the parameters of unrecorded regions. Finally, a monosynaptic glutamatergic projection from frontal cortex to the STN constituted the hyperdirect pathway.

Recent work has emphasized the significance of the hyperdirect pathway for the functional organisation of cortico-basal ganglia-thalamocortical circuits [30] and, importantly, this pathway has been shown to be crucial for the expression of abnormal slow oscillations in the STN-GPe network in Parkinsonism [33]. In short, our standard model architecture incorporates the major glutamatergic and GABAergic connections between the six key components of the cortico-basal ganglia-thalamocortical circuit. In accommodating the core elements of the loop circuit, the model also adheres to the established organisational principles embodied in the direct, indirect and hyperdirect pathways. Note that our standard model does not include all known connections. However, the addition of more connections does not necessarily improve the ability of the loop circuit (and model) to sustain beta oscillations. To test this, we tried adding two less well-studied, but potentially important, pallidofugal connections, either from GPe to EPN or that from GPe to striatum [34]. The addition of either connection did not improve the performance of (evidence for) the standard model, which provided the optimum balance of accuracy and complexity for our given data set (see Figure S2 in Text S1).

### Beta Oscillations and Effective Connectivity

A time-frequency analysis of resting state LFPs at 10–35 Hz revealed consistent and long-lasting high-amplitude beta oscillations in all cortical and BG recordings from the 6-OHDA-lesioned animals (*n* = 9 rats; Figure 1B). However, no dominant band-limited LFP activity was observed in control animals with intact dopamine (*n* = 8, Figure 1B). Since the spectral characteristics remained constant over time, we assumed stationarity in our recordings and characterised that steady-state behaviour using their cross spectra. Using the cross spectral densities, we inverted the DCM for each animal individually and for each group's average spectral response. Model inversion entails estimating the mean and variance of the unknown model parameters 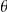 that summarise their posterior or conditional density 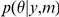 conditioned upon the cross-spectral density data-features 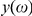 and the model 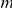 described above. These unknown parameters include the biophysical parameters of the neural-mass model as well as parameters controlling the spectral composition of neuronal and channel noise. These noise parameters control a mixture of white and pink noise assumed to exist at BG and cortical channels separately (see Materials and Methods and [20]). The priors on these unknown parameters 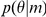 used standard values (see Table 1 in Text S1).

**Figure 4:**
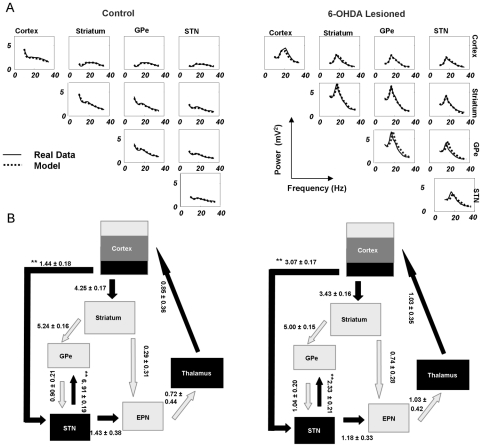
Model fits and posterior connectivity estimates. (A) Observed and modelled cross-spectral densities. Densities for frequencies from 10 Hz to 35 Hz were extracted from time domain data using a vector autoregressive model. *Left* Average LFP data (full lines) and average DCM fits (dashed lines) for control animals (n = 8). The main diagonal displays the auto-spectral densities at each electrode and the off-diagonal elements display the two-way cross spectra. *Right* Average data and model fits in Parkinsonian animals (n = 9). Note that only the Parkinsonian animal data and model fits have prominent beta-band peaks. (B) Posterior connectivity estimates from each individual animal's DCM were combined to form group averages. Average MAP estimates are shown with 95% Bayesian credible intervals. *Left* Average connectivity estimates from the control animals. The strongest connectivity in this circuit is the excitatory connection from STN to GPe. Similarly strong connections exist for cortical connections to the striatum and for striatal connections to the GPe. *Right* Posterior estimates from the 6-OHDA-lesioned animals are also strong along the indirect pathway from striatum to GPe and at cortical efferents to striatum and STN. Differences in connectivity strengths between the control and Parkinsonian groups with a probability >99.99%, are indicated**. Two key differences are observed. *1.* the hyperdirect pathway is stronger in 6-OHDA-lesioned animals, and *2.* STN input to GPe is weaker in lesioned animals.

**Figure 5:**
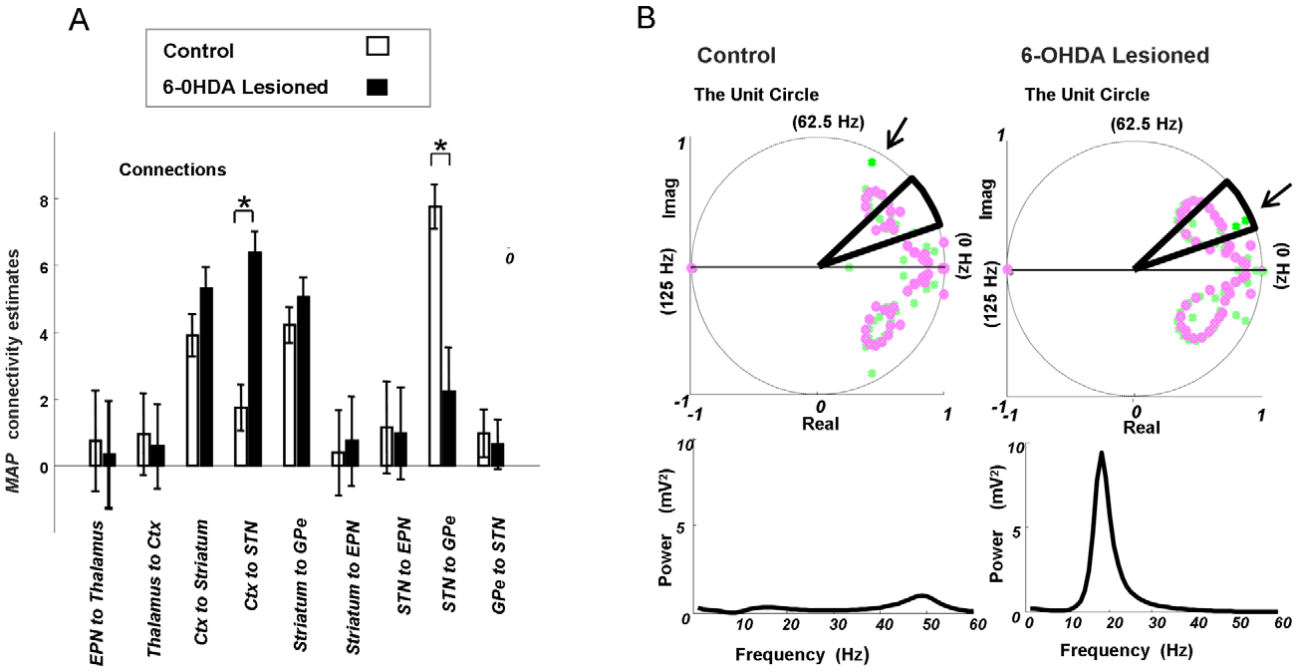
Average data DCMs and pole zero representation of the system. (A) MAP estimates of extrinsic connectivity parameters (comparable to Figure 2B) but for the DCMs optimised for the grand averaged control and lesioned data. Differences in connectivity strengths (with a posterior probability >95%) are denoted with an asterix. As shown above in the individual animal DCMs, the hyperdirect pathway is stronger in Parkinsonian animals and STN to GPe connectivity is weaker. (B) Simulated system responses using the posterior estimates from the two grand averaged spectra DCMs (Figure 3A). *Top Left* Pole zero representation of control DCM. Poles (*green x*) reflect points of infinite power and zeros (*magenta o*) are points of zero power. The unit circle drawn along a real axis and imaginary axis (Imag) delimits a stable (poles within) or unstable (poles without) system impulse response, where the response is symmetrical with respect to the real axis. The response from 0 Hz to the Nyquist rate (125 Hz) is represented along the unit circle from (1, 0) to (−1, 0) in the positive imaginary plane and the response from 0 Hz to −125 Hz is represented from (1, 0) to (−1, 0) in the negative imaginary plane. Frequencies along the unit circle that are close to poles are prominent in the system's output. The system response around beta frequencies is demarcated on the circle using black solid lines (13–30 Hz), to the left of this quadrant contains gamma oscillations (30–125 Hz). We note that the bilinear transform used to obtain the z-domain characterisation introduces a frequency warping where nonlinearities result in 13–30 Hz being mapped to 12.89–28.68 Hz on the unit circle. In the case of the control animals, a pole close to 50 Hz (highlighted with an arrow) leads to a transfer function (*Below Left*) with a small spectral peak in the gamma band. In the case of the 6-OHDA-lesioned group (*Right*) two poles close to 20 Hz (highlighted with an arrow) lead to a high amplitude beta peak in their transfer function. The input–output characteristic of the systems are illustrated using cortical input and STN output pair.

In Figure 4A we plot the predicted and observed magnitudes of the cross-spectra averaged over animals in control and lesioned groups. The model fits show that the DCMs reproduce the key spectral properties of LFPs recorded in both animal groups: The LFPs from the control animals contain relatively low power across a broad band of frequencies captured by the control DCMs, while the LFPs in Parkinsonian animals contain a high-amplitude beta band peak (average peak frequency of 17 Hz), which in turn was captured by the Parkinsonian DCMs. Differences in effective connectivity underlying these spectra were then examined using Bayesian parameter averaging [35]. Figure 4B shows the connection strengths averaged over individual estimates in each group. These mean or *maximum a posteriori* (MAP) estimates are plotted with 95% Bayesian confidence intervals. We considered connections changed when the probability of a difference was greater than 99.99%. Along the hyperdirect pathway, cortical output to the STN increased in the Parkinsonian animals compared to the control group while, conversely, efferent connections from the STN to GPe decreased. These changes in effective connectivity, occurring after dopamine cell lesions, characterise the circuit generating beta oscillations and the net balance between pathogenesis and any consequent compensatory changes. The DCMs of the grand averaged spectral responses (where one DCM was fit to the average control cross-spectra and one DCM was fit to the average lesioned cross-spectra) confirm the inference based on individual DCMs (Figure 5A), where the same differences in MAP estimates i.e. the connection strengths subtending the average control and Parkinsonian data, were found for the hyperdirect connection and STN to GPe connection. We examined the posterior correlations among parameters from these DCMs to preclude identifiability issues: Where high dependencies exist between two connections, a change in either could account for the same data. We show however that this is not the case among our parameters of interest (extrinsic connectivity measures) where, on average, only small correlations (∼0.1) were seen (see Figure S7 in Text S1). We also examined the robustness of lesion-related changes in the circuit by removing the STN to GPe connection and looking for a difference between control and Parkinsonian networks: We again found an increase in hyperdirect connectivity for the Parkinsonian model (see Figure S8 in Text S1), even in the absence of changes in STN to GPe connectivity.

Using the posterior estimates from the DCM of the grand averaged spectra, we simulated the system's response for a wide band of frequencies. This allowed us to identify the predominant changes in spectral activity, associated with our optimised models of control and Parkinsonian animals: For linear systems, the frequency response can be illustrated as poles and zeros on the unit circle. This involves a reformulation of the system (i.e. the differential equations used by the DCM) using a z-transform [21]. This transform produces a transfer function, which summarises the model system's input-output spectral properties.

In Figure 5B, we show the average input-output characteristics of the model by simulating cortical input and illustrating STN output. In the circuit based on the control group, we observe a pole close to the unit circle at around 50 Hz (a pole is a point of infinite system response) thereby producing a spectral peak at this gamma frequency in the transfer function. This gamma feature has been consistently reported in LFPs recorded from the basal ganglia in alert, dopamine-intact animals [36,37,38]. Crucially, our generative model captured this, even though it was only optimised using LFP data over 10–35 Hz. In light of this finding we constructed new spectral estimates from the original data for frequencies from 40–80 Hz and confirmed a prominent spectral peak in the gamma band at BG probes for control animals (see Figure S3 in Text S1). This finding highlights the predictive validity of our model. In contrast, in the Parkinsonian circuit, we found two poles near the unit circle, at around 20 Hz, which produced a high-amplitude spectral peak at beta frequencies.

### Exacerbating Beta Oscillations

**Figure 6:**
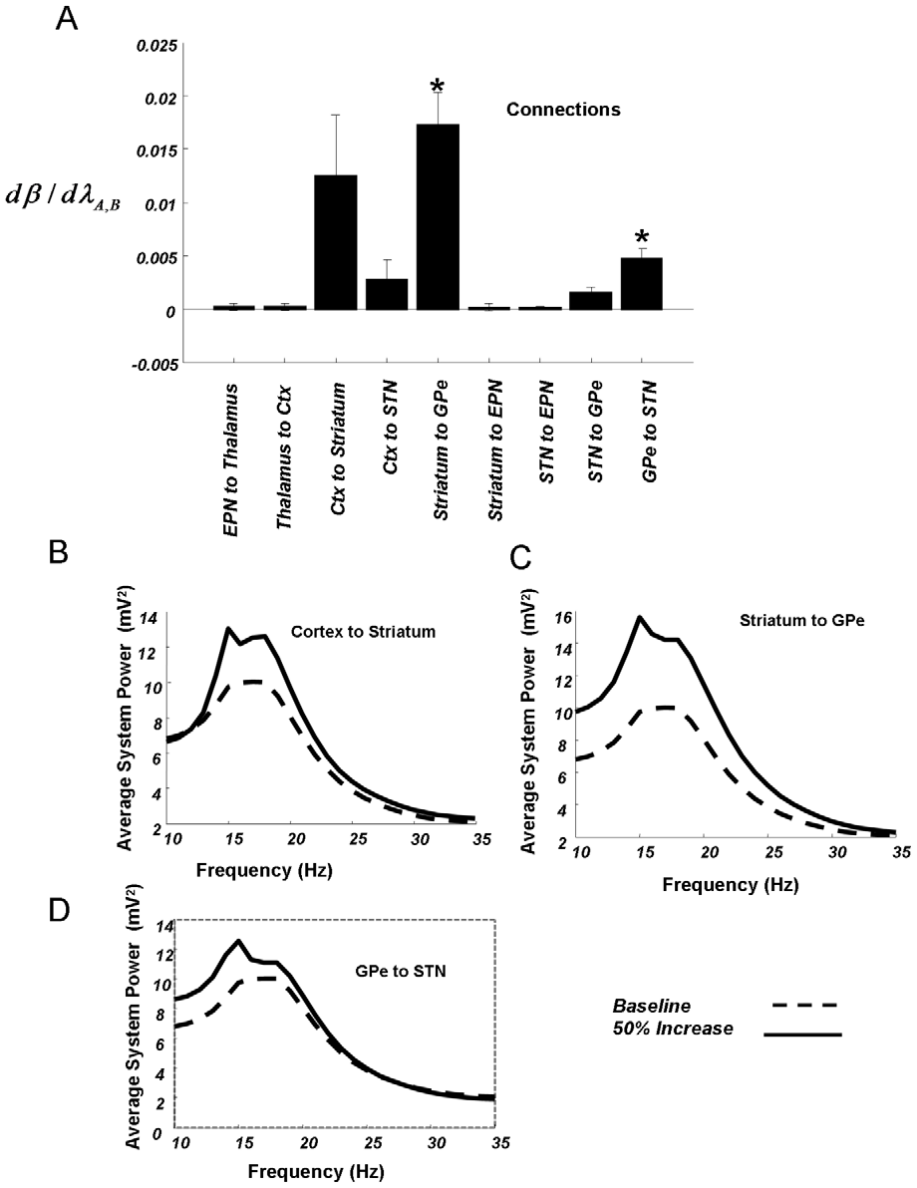
Contribution analysis. (A) Using the DCMs from the Parkinsonian animals, we quantified changes in beta power with respect to changes in connectivity parameters (averaged over channels). The measure of beta power was the height of the beta peak centred at 17 Hz (summed over 16–18 Hz). Increasing connections from striatum to GPe and from GPe to STN exacerbated beta oscillations (positive derivatives), where the striatum to GPe connectivity was the most effective (*p<0.05 Bonferroni corrected for 9 multiple comparisons; Error bars denote s.e.m.). (B*)* The average spectral response (over animals and circuit nodes) is plotted using baseline parameter values (dashed line) and an increase in cortex to striatum of 50% (solid line). (C) The same baseline transfer function, with altered transfer function following a 50% increase in connection strength from striatum to GPe. (D) Baseline and altered transfer functions following a 50% increase in GPe to STN (See also see Figures S4, S5 and S6 in Text S1).

The Parkinsonian circuit we have described represents the effects of chronic dopamine depletion and, potentially, a balance between primarily pathogenic changes and compensatory or adaptive changes. Consequently, we asked which connection strengths in the reorganised Parkinsonian circuit could contribute to (or attenuate) beta activity. This entailed using the optimised DCM from the lesioned animals to predict spectral responses to changes in the model circuit architecture: To do this, we quantified the degree to which a change in each connection affected beta activity throughout the circuit (power summed over 16–18 Hz and channels). This provided a measure of ‘beta contribution’ per animal, per connection; in terms of the partial derivative of summed beta activity, with respect to each connection. Note that beta contributions (derivatives) could be either positive, whereby small changes exacerbate beta, or negative whereby small changes ameliorate beta (e.g. see Figures S4 and S6 in Text S1). Using each animal's average contribution (see Equation 10) as a summary statistic, we performed a one-way ANOVA with connection as a factor, and revealed a significant effect of connection (*F*(8,72)  = 7.42, *p<0.0001;* Figure 6A). *Post hoc* two way *t*-tests (to cover positive and negative derivatives), indicated that the beta contributions were significant for two connections when corrected for multiple comparisons. We found that variations in striatal connections to GPe (*p<0.05* Bonferroni corrected) and GPe connections to STN (*p<0.05* Bonferroni corrected) produced significant increases in beta power (Figure 6A). These tests rest on the consistency of these effects over animals, whereas considering their magnitude alone, would suggest a strong beta contribution from cortex to striatum. Thus incremental increases in connections along the so-called indirect pathway [2] consistently exacerbated beta oscillations across Parkinsonian animals. This was also seen in terms of the magnitude of the effect on beta oscillations across animals (Figure 6A). The three connections with the highest average beta contribution were cortex to striatum, striatum to GPe and GPe to STN. To illustrate these effects we computed the system transfer function using a gain of 50% in these connection strengths. Figure 6B displays the transfer function averaged over nodes and animals at baseline levels and with the cortex to striatum connection increased by 50%. Figure 6C displays the same graphs but now for baseline levels and with striatum to GPe increased by 50%. Similarly in Figure 6D we show baseline and altered transfer functions using a 50% increase in connection strength from GPe to STN. Overall findings were similar when we repeated our analyses using independent data from the same animals (see Figure S4 in Text S1).

These simulations suggest that if connections along the indirect pathway could be weakened in the Parkinsonian state, then excessive beta-activity might be attenuated, thereby providing important candidate therapeutic targets. An apparent paradox is that while we find an increase in effective connectivity in the hyperdirect pathway from cortex to STN, our contribution analysis shows that manipulating the strength of this connection did not change beta activity significantly. Conversely, the remaining two connections (striatum to GPe and GPe to STN) that profoundly influenced beta activity in our simulations did not differ in their mean strength between the control and Parkinsonian groups. This apparent paradox can be resolved by considering the extended network that incorporates all nine connections. The strength of a connection need not necessarily change between the healthy and diseased states in order for that connection to have a different effect on oscillatory strength within the new network; in other words, perturbations to a connection with a given strength will have a different functional effect, depending on the network in which it is embedded. Moreover since the conditional densities of the parameters of interest in the DCM are identifiable (see Figure S7 in Text S1) we can surmise an effect along the indirect pathway connections.

We have clear evidence that the extended network differs between the control and Parkinsonian states with increases in the effective connection strength of the hyperdirect cortex to STN pathway and reduction in that from STN to GPe. Above, we identified that several connections in the indirect pathway have the capacity to dynamically modulate beta. Thus we compared beta contribution between the control and Parkinsonian animals for the ‘beta critical’ indirect pathway connections. This showed that these connections engendered much higher beta activity when embedded in the Parkinsonian network (two sample t-test; *p<0.05*; see Figure S5 in Text S1), even though their strengths *per se* did not differ significantly between the healthy and disease states. Furthermore, we tested the specificity of the indirect pathway's role and repeated the contribution analysis for the gamma band (at 60 Hz). This analysis showed that the frequency promotion in the Parkinsonian state was specific to the beta band and could not be generalised to higher frequencies (see Figure S6 in ).

## Discussion

Classical models of connectivity within the cortico-basal ganglia-thalamocortical loop circuit explain PD symptoms in terms of altered firing rates along the direct/indirect pathways [2]. More recent research has highlighted the oscillatory nature of excessive neuronal synchronisation in the Parkinsonian state [7]. Here, we present a new model of effective connectivity in the cortico-basal ganglia-thalamocortical loop, which emphasizes neuronal synchrony and oscillatory dynamics over rate coding. Our scheme is based on dynamic causal modelling of multisite LFPs, which, in order to be detected, necessitate spatiotemporal summation and hence, synchronisation of population activity. Importantly, the LFP data were derived from a rat model of PD that recapitulates clinical pathophysiology, most notably the dominance of beta oscillations in the untreated Parkinsonian state. Using neural mass models that comprise ensemble firing output and membrane potential inputs [27], our model can generate low-frequency broadband activity or Parkinsonian excessive beta activity by increasing and decreasing particular extrinsic connections. In effect, operating from a stationary equilibrium, the Parkinsonian cortico basal-ganglia-thalamocortical circuit has a modulation transfer function that peaks at beta frequencies. Similar frequency tuning is seen in the cortico-basal ganglia circuit of untreated PD patients in response to phasic inputs to STN [39].

We found specific differences between control and Parkinsonian groups at two pathways along re-entrant circuits. The effective connection strength of the cortical ‘hyperdirect’ input to the STN was dramatically increased in the Parkinsonian animals, compared to control animals, a finding in accord with current views of subthalamic hyperactivity in PD [5,6,33,40], and also with optogenetic circuit perturbations that point to the cortex as a key driver of this hyperactivity [17]. In contrast, the STN input to the GPe decreased in the Parkinsonian animals. The latter represents a potentially important and novel finding. Nevertheless, as discussed below, it builds on a literature implicating the reciprocally-connected STN-GPe network in the generation and dissemination of abnormally synchronized oscillations in Parkinsonism [5,41,42].

Our standard model architecture might not be the only one that can sustain exaggerated beta oscillations. However, our model does incorporate the major glutamatergic and GABAergic connections between the six key components of the loop circuit, thus capturing the core elements of the direct, indirect and hyperdirect pathways and placing it within established frameworks. The architecture was chosen as the simplest that could support an answer to our questions about inter-regional connectivity. The large parameter space and the effective data size (via the AR spectral decomposition) would normally predispose to ‘over fitting’ in a maximum likelihood setting. However, Bayesian inference finesses this problem because it optimises the marginal likelihood (model evidence), which includes a complexity term. This complexity rests on the use of priors or constraints on the underlying neurobiology (e.g., synaptic time constants). This effectively limits the degrees of freedom in the model to those parameters with relatively uninformative priors; i.e., the connectivity parameters of interest. Moreover, the standard model performed no better when two additional pallidofugal connections were incorporated (see Figure S2 in Text S1). Importantly, the model was successful in making valid predictions regarding frequencies not considered during model inversion. These predictions, namely greater gamma-frequency activity in control animals compared to the Parkinsonian state, were corroborated in a separate analysis (see Figure S3 in Text S1). Together this prediction and subsequent analysis lends weight to the assumption of a biologically plausible and useful model.

The documented changes in steady-state effective connectivity may indicate primary pathological changes and/or secondary compensatory mechanisms (see Figure S1 in Text S1). Importantly, as circuit changes are delayed following chronic dopamine depletion [6,8], it is possible that some changes in effective connectivity represent long-lasting changes that might not be amenable to acute reversal with dopamine, while others might be more dynamic. In this regard, it is interesting that although the hyperdirect pathway was strengthened in the Parkinsonian state, changing its connection strength thereafter in the contribution analyses had relatively little effect on the dynamics of the reorganised system as a whole (see Figure S1 in Text S1). This suggests that the strengthening of the hyperdirect pathway may be a necessary, permissive, chronic plastic change for the larger circuit to become susceptible to pathological beta oscillations, but thereafter has relatively little dynamic influence, at least over the short-term.

The decrease in STN input to the GPe in Parkinsonian animals is also interesting. Here, contribution analyses demonstrated that increasing the strength of the reciprocal connection exacerbates the beta oscillations that characterise the Parkinsonian state. Recent experimental data also suggest that interactions between GPe and STN could both support and actively promote the emergence of excessively synchronized oscillations at the network level [5], while a recent computational model also emphasises the importance of strong excitatory connections from STN to GPe in the promotion of beta-frequency activity [42]. The reduction in STN to GPe connectivity that we observed here may instantiate compensatory neural plasticity (see Figure S1 in Text S1), acting to limit reciprocal feed-back from GPe to STN, thus ameliorating Parkinsonism.

The above findings underscore the importance of our contribution analysis in interpreting the nature of steady-state changes. It is important to note that while the effects found in our contribution analysis are dependent on the model inversion as a whole, i.e. including intrinsic parameters, it is the change in the extrinsic connections themselves that promote beta oscillations. This approach was key to identifying the indirect pathway connections as influencing abnormal activity in the chronicly reorganised circuit, even though their connection strengths remained relatively unchanged between control and Parkinsonian states. The importance of these connections could not have been suspected from simple contrasts of control and Parkinsonian steady-state networks, and it was their potency in promoting beta oscillations that was very much greater when embedded in the Parkinsonian network. This means that connections of the indirect pathway have a new strategic role in the re-organised circuit, and provide potential therapeutic targets, in line with the recent finding that selective excitation of striatal neurons in the indirect pathway elicits a Parkinsonian behavioral state [43]. Indeed, it is likely that D2-mediated suppression of striatal input to GPe might explain the attenuation of beta oscillations in patients with PD following therapy with apomorphine [44] or L-Dopa [10].

Our results should encourage exploration of the therapeutic potential of suppressing the GPe to STN connection in PD. The directionality of this prediction is important given the different neurotransmitters in reciprocal STN-GPe connections. We have assumed that the network changes occurring under anaesthesia are also relevant in the awake, behaving animal. There is good evidence to support an extrapolation of our findings to the un-anesthetized state. First, the excessive beta oscillations we have modelled occur in both anesthetized and awake 6-OHDA-lesioned rats [5,6,8,22], moreover the gamma activity shift for the control animals reveals how broadband spectral activity is preserved under anaesthesia. Second, at least some of the network alterations defined here are likely the result of chronic plasticity as they only appear several days after dopamine neurons are lesioned [5,8]. Thus, some of the critical features identified in the model fitting from the anesthetized state are likely to represent underlying changes in the microcircuit, and therefore may still be relevant in the awake state.

In summary, our analyses lead to a new view of connectivity in the cortico-basal ganglia-thalamocortical circuit, which acknowledges the importance of synchrony in the pathophysiology of Parkinson's disease [7]. Our scheme makes strong and testable inferences about what are essentially permissive vs. compensatory changes as well as which connections have altered strategic contributions to the pathological state. These connections represent candidate therapeutic targets (see Figure S1 in Text S1). Key amongst the latter are connections to and from the GPe [40,45].

## Materials and Methods

### Electrophysiological Recordings

Experimental procedures were carried out on adult male Sprague-Dawley rats (Charles River, Margate, UK), and were conducted in accordance with the Animals (Scientific Procedures) Act, 1986 (UK). Recordings were made in eight dopamine-intact control rats (288–412 g) and nine 6-OHDA-lesioned rats (285–428 g at the time of recording), as described previously [5,6,46]. Briefly, anaesthesia was induced with 4% v/v isoflurane (Isoflo™, Schering-Plough Ltd., Welwyn Garden City, UK) in O_2_, and maintained with urethane (1.3 g/kg, i.p.; ethyl carbamate, Sigma, Poole, UK), and supplemental doses of ketamine (30 mg/kg, i.p.; Ketaset™, Willows Francis, Crawley, UK) and xylazine (3 mg/kg, i.p.; Rompun™, Bayer, Germany). The electrocorticogram (ECoG), a type of cortical local field potential, was recorded via a 1 mm diameter steel screw juxtaposed to the dura mater above the right frontal (somatic sensory-motor) cortex (4.5 mm anterior and 2.0 mm lateral of bregma [47] and was referenced against another screw implanted in the skull above the ipsilateral cerebellar hemisphere. Raw ECoG was band-pass filtered (0.3–1500 Hz, −3 dB limits) and amplified (2000×; DPA-2FS filter/amplifier: Scientifica Ltd., Harpenden, UK) before acquisition. Extracellular recordings of LFPs in the striatum, GPe and STN were simultaneously made in each animal using ‘silicon probes’ (NeuroNexus Technologies, Ann Arbor, MI). Each probe had one or two vertical arrays of recording contacts (impedance of 0.9–1.3 MΩ measured at 1000 Hz; area of ∼400 µm^2^). The same probe was used throughout these experiments but it was cleaned after each experiment in a proteolytic enzyme solution to ensure that contact impedances and recording performance were not altered by probe use and re-use [33]. Monopolar probe signals were recorded using high-impedance unity-gain operational amplifiers (Advanced LinCMOS: Texas Instruments, Dallas, TX) and were referenced against a screw implanted above the contralateral cerebellar hemisphere. After initial amplification, extracellular signals were further amplified (1000×) and low-pass filtered at 6000 Hz using programmable differential amplifiers (Lynx-8: Neuralynx, Tucson, AZ). The ECoG and probe signals were each sampled at 17.9 kHz using a Power1401 Analog-Digital converter and a PC running Spike2 acquisition and analysis software (Cambridge Electronic Design Ltd., Cambridge, UK). Neuronal activity was recorded during episodes of spontaneous ‘cortical activation’, which contain patterns of activity that are similar to those observed during the awake, behaving state [48]. Cortical activation was defined according to ECoG activity [5,6]. Neuronal activity patterns present under this anaesthetic regime may only be qualitatively similar to those present in the unanesthetized brain. However, the urethane-anesthetized animal still serves as a useful model for assessing ensemble dynamics within the basal ganglia [46]. Indeed, in 6-OHDA-lesioned animals, exaggerated beta oscillations emerge in cortico-basal ganglia circuits during activated brain states [5,6] thus accurately mimicking the oscillatory activity recorded in awake, unmedicated PD patients [10].

### 6-Hydroxydopamine Lesions of Dopamine Neurons

Unilateral 6-OHDA lesions were carried out on 200–250 g rats, as described previously [5,6]. Twenty five minutes before the injection of 6-OHDA, all animals received a bolus of desipramine (25 mg/kg, i.p.; Sigma) to minimize the uptake of 6-OHDA by noradrenergic neurons [49]. Anaesthesia was induced and maintained with 4% v/v isoflurane (see above). The neurotoxin 6-OHDA (hydrochloride salt; Sigma) was dissolved immediately before use in ice-cold 0.9% w/v NaCl solution containing 0.02% w/v ascorbate to a final concentration of 4 mg/ml. Then 3 µl of 6-OHDA solution was injected into the region adjacent to the medial substantia nigra (4.5 mm posterior and 1.2 mm lateral of bregma, and 7.9 mm ventral to the dura [47]. The extent of the dopamine lesion was assessed 14–16 days after 6-OHDA injection by challenge with apomorphine (0.05 mg/kg, s.c.; Sigma [50]). The lesion was considered successful in those animals that made >80 net contraversive rotations in 20 min. Note that the emergence of exaggerated beta oscillations after 6-OHDA lesions is not dependent on apomorphine [6,22]). Electrophysiological recordings were carried out ipsilateral to 6-OHDA lesions in anesthetized rats 21–42 days after surgery, when pathophysiological changes in the basal ganglia are likely to have levelled out near their maxima [6].

### Data Pre-Processing: Evaluating Cross-Spectral Densities

We restricted our analysis to the ECoG and LFP activities present during a spontaneous activated brain state, which mimics that accompanying alert behaviours in which beta oscillations are most prominent in PD patients and 6-OHDA-lesioned rats [5,6,22,51]. Thirty second epochs of cortical activation were analysed from one contact in each BG nucleus and the contemporaneous ECoG from each animal. Cross-spectral density 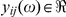 between channels *i* and *j* (with *i*:1,...4 and *j*:*i*,..4) were evaluated from the channels' time-series, 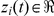 using the following two steps (Equations 1 and 2). Note that although 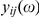 are the data features predicted by the model, we first estimate these features from the recorded time-series.

Data from the four channels (Cortex, Striatum, GPe and STN) were summarised using an autoregressive (AR) process [52] of order 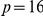. This order determines the number of peaks (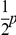) in the associated spectra [53] and was chosen to approximate the asymptotic order of the neural mass model investigated in Moran et al. [20]. 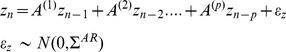
(1)Here, 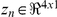 is a column vector containing data samples from the four channels at time *n,* 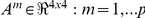 is a matrix of AR coefficients (weights), and 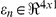 is a random noise term, assumed to be sampled from a zero mean Gaussian with covariance 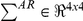. Given 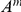 and 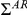, the cross-spectra 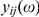 between channels *i* and *j* can then be constructed from the (complex) transfer functions 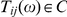 using standard linear systems theory:
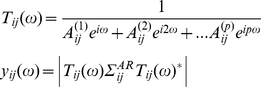
(2)Here, we used 26 frequencies 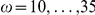 Hz at 1 Hz resolution. The cross-spectra were estimated from Equations 1 and 2 using a variational Bayesian algorithm, [52] (as implemented in the spectral toolbox of http://www.fil.ion.ucl.ac.uk/spm/). These cross-spectra were then used for dynamic causal modelling:

### Dynamic Causal Modelling

DCM is a model comparison framework for the inversion and comparison of generative (forward) models based on differential equations. DCM allows data from multiple recording sites to be analysed as a distributed system. Originally developed for fMRI [54], the framework uses a generative model of the neural processes (usually neural mass and mean field models) [55,56] that cause observed data. Bayesian model inversion furnishes estimates of coupling or effective connectivity between regions and how this coupling is changed by experimental context [57]. For LFP and ECoG data, the generative model contains details about the structure and synaptic properties within neuronal sources, as well as the synaptic input that each source receives [21]. In what follows, we describe the precise form of the dynamic causal model we used in this application. The underlying neural mass model has been described and validated in a series of previous papers [20], [21], [28] and is briefly reprised below for completeness.

In DCM for steady-state responses, one is trying to predict or explain observed spectral activity. Effectively, this entails modelling the mapping between the spectral density of neuronal fluctuations or innovations and the resulting responses. This mapping is parameterised in terms of the (synaptic) parameters of a neural mass model. The neural mass model is used to determine how random neuronal fluctuations are filtered to produce observed cross-spectra. Model inversion involves optimising model parameters to explain empirical cross-spectra. In what follows, we will describe the generative model in terms of the kernels or transfer functions that couple the spectra of neuronal innovations to observed cross-spectra and then describe the neural mass model that determines how the transfer functions (kernels) are parameterised. Figure 2 provides a summary of the generative model in terms of a Bayesian Network, while Figure 3 describes the neural mass model that provides the kernels in Figure 2.

### The Likelihood Model

We require the model to predict cross-spectra corresponding to the data features described above. This prediction is based on the parameters 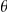 of some model of how these spectra were generated. We assume the observed cross-spectra are a mixture of predictions and Gaussian error
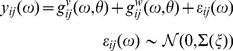
(3)The spectral prediction 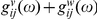 (obtained from the Fourier transform of the time domain dynamics, Equations 4 & 5) comprises two parts: The first corresponds to cross-spectra due to neuronal activity, while the second, 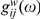 corresponds to cross spectra induced by channel noise 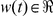 (described below). The error 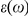 has a covariance matrix 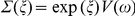 where 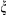 are unknown covariance parameters and 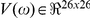 encodes correlations over nearby frequencies. The exponential transform ensures the error covariance is positive definite. [We note that cross spectral densities will asymptote to a Wishart distribution at a large sample limit [58]. However, when averaging each cross spectrum over multiple trials, one can appeal to the central limit theorem and assume a normal distribution for the differences between observed and predicted cross spectra (see [59] for a comprehensive treatment)]. Assumptions about the observation error allow one to compute the probability of obtaining some data, given the parameters, this is called the likelihood model (see below). Channel or instrumentation noise was modelled separately for the cortex (ECoG electrode) and BG to account for differences in electrode size between the silicon probes and the ECoG screw. Furthermore, we modelled common BG noise components due to volume conduction in the BG. The ensuing predictions are given by standard linear systems theory:
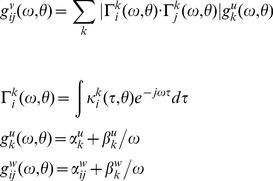
(4)Here 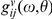 is the sum of cross-spectral densities induced by the inputs or neuronal innovations 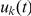 driving neuronal dynamics. These cross-spectra are simply the (complex) transfer functions 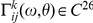 mapping from the *k*-th neuronal innovations (*k*: 1,..5) to each channel times the spectral density of each input: 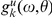. We parameterised the spectra of the neuronal innovations and the channel noise as a mixture of white and pink components [60,61].

The transfer functions are the Fourier transforms of the corresponding first-order kernels, 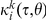 that mediate the effect of the *k*-th innovation (zero mean fluctuations, 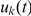, which we assume perturb the system linearly around its fixed point) on the observed data. These kernels can be regarded as impulse response functions of the *i*-th channel to the *k*-th input: i.e., the change in output with respect to a change in input at time 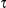 in the past. First order kernels are ubiquitous representations of dynamical systems, where the response (of linear systems) can be determined by convolving the input with the system's impulse response or kernel. In the frequency domain, where convolution becomes multiplication, multiplying the Fourier transform of the kernel with the Fourier transform of the input provides the system's spectral response: c.f. Equation 4 (for an introduction to these transforms, see [62]). The kernel for each channel obtains analytically from the Jacobian 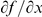 of the flow or motion 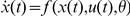 of hidden neuronal states specified by a neural mass model (see below). This flow describes how the hidden neuronal states are perturbed by the inputs or innovations. For channel *i*, and input *k* the kernel is
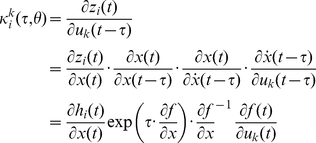
(5)This means the kernels are analytic functions of the equations of motion of the hidden states, the mapping between hidden states and inputs. In addition, we have to model the mapping between hidden states and observed channel data in the time domain: 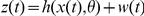. This time domain prediction forms the basis of the spectral prediction through Equations 4 and 5. The observation function 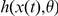 used here was a simple mixture of depolarisations at pyramidal, stellate and inhibitory interneurons contributing to the cortical ECoG channel and the depolarization of the individual cell populations in each BG recording. The ECoG data are assumed to arise predominantly (60%) from the pyramidal cells due to their dendritic organisation [63], with a 20% contribution from the net membrane potentials of the inhibitory interneurons and stellate cells [20]). These assumptions are encoded in the priors on the parameters of the observation function described later.

### The Neural Mass Model

Equation 5 allows us to predict the systems kernels and spectral behaviour given the equations of motion (differential equations) that constitute our model of hidden neuronal states. The effect of neuronal noise or fluctuations is modelled here in terms of their expression as cross spectra in channel space. The neural model is composed of subpopulations, where each subpopulation can have different synaptic rate constants and amplitudes. Subpopulations are grouped into sources and coupled with intrinsic connections, while sources are connected by extrinsic connections between specific subpopulations in different sources. Our model comprised a cortical source, four basal-ganglia nuclei and a thalamic source (Figure 1A). The cortical source contained three neuronal subpopulations (two excitatory and one inhibitory, [20]), while the basal ganglia and thalamic nuclei contain one subpopulation, whose afferents are each either excitatory or inhibitory, according to their known neurochemistry [29] (Figure 3). The major glutamatergic and GABAergic connections between the six key components of the cortico-basal ganglia-thalamocortical circuit comprise our standard model architecture.

The dynamics of hidden neuronal states and the ensuing observation are assumed to have the form
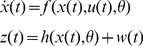
(6)Where 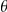 are unknown biophysical parameters of the model of neuronal dynamics. These dynamics are modelled in terms of the evolution of voltages and currents in each subpopulation, 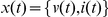
[64]. This evolution rests on two operators: The first transforms presynaptic inputs (firing rates) into a postsynaptic membrane potential 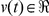 response. This is modelled by convolution with a synaptic impulse response (alpha) function, where the synapse is either inhibitory or excitatory [28,64,65,66]. The second operator is a nonlinear function of postsynaptic depolarisation that returns the firing rate. This firing rate is passed to other subpopulations though intrinsic (within source) and extrinsic (between source) connections to provide presynaptic input. The synaptic convolutions can be formulated as ordinary differential equations describing postsynaptic currents 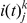 and voltages 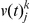 for the *j*-th subpopulation in the *k*-th source:
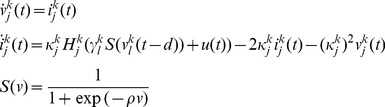
(7a)This can be expressed more compactly in terms of the second order differential equation in voltage:
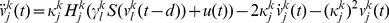
(7b)The parameters 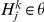 control the amplitude of the response function of the *j*-th subpopulation in the *k*-th source, while and 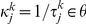 are lumped representations of passive membrane rate (inverse time) constants. Here, 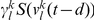 represents presynaptic input, which is a sigmoid function (with slope parameterised by 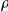) of delayed depolarisation in the *l*-th subpopulation (*l*: 1,..3 in cortex and *l*: 1 in the Basal Ganglia), weighted by an intrinsic coupling parameter 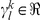. For simplicity, we have omitted extrinsic inputs from the *i*-th source: 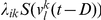, which have a similar form (please see Figure 3) but have included neuronal noise or fluctuations, 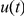. Extrinsic delays between sources are parameterised by 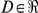 and intrinsic delays between cortical layers by 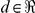.

The architecture depicted in Figures 1 and 3 outlines the cell types within each neural-mass or ensemble and connections between ensembles. Of interest here are the extrinsic connection parameters 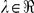, which scale the influence of firing rate from different sources. As noted above, the parameters 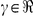 encode the strength of intrinsic connections between cortical layers. This gives the real positive parameter set 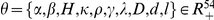 **,** where 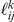 are the coefficients (electrode gains) of the observation function 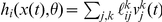. These determine the contribution of the depolarisation 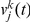 to the *i*-th electrode, as described above.

### The Priors

Because the model parameters are non-negative they are treated as scale-parameters with Gaussian priors on the log of their values. These are specified in terms of a prior mean 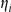 and variance 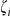 for the *i*-th parameter. A non-zero prior variance allows the parameter to be pushed from its prior mean. A relatively tight or informative prior obtains when 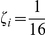 (see Table 1 in Text S1) and with typical data, this allows a re-scaling of the posterior mean around the prior mean by up to a factor of about two. Relatively flat priors, like those used for our key parameters of interest; the effective connectivity measures, allow for an order of magnitude scaling (with a prior variance of 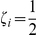). The maximum excitatory amplitude and time constant had prior means of 8 mV and 4 ms respectively, while the inhibitory parameters have prior means of 32 mV and 16 ms [67]. These synaptic parameters have a prior variance of 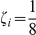, allowing for a scaling up to a factor of about four ([56], see Table 1 in Text S1). As noted above, the connectivity parameters have a higher prior variance than the other biophysical parameters. This ensures their posterior estimates are determined primarily by the data. The priors over the parameters, 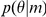 are detailed in Table 1 in Text S1 and Moran et al. [20]. Finally, we used non-informative priors 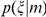 over the covariance scale-parameters.

### Model Inversion

The priors, together with the likelihood model (Equation 3), constitute a generative model of observed cross spectral densities based on a neuronal state-space model formulated in continuous time. This generative model is summarised in Figure 2 and can be expressed in terms of the joint density over the spectral data and model parameters
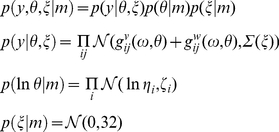
(8a)The parameters of interest are the effective connectivity from one subpopulation to another. Note that the hidden neuronal states are not estimated explicitly in DCM for steady state responses, because we can map directly from cross spectrum of neuronal innovations to observed cross spectra, using linear systems theory. To invert the model, we seek the moments of the posterior probability distribution 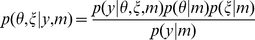
(8b)However, this expression contains a normalisation constant (the model evidence) which would require the intractable integral calculation 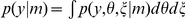 (recall that 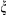 parameterises the variance of observation error). We therefore employ a variational scheme to approximate the solution to Equation 9, using a lower bound on model evidence as an objective function [68]. Maximising this objective function returns the maximum *a posteriori* (MAP) parameter estimates (the conditional or posterior mean) and the conditional covariance. This scheme is known as Variational Laplace [57] and grandfathers most model inversion and Bayesian filtering schemes, under Gaussian assumptions:

### Variational Laplace

Variational Laplace appeals to a mean field approximation and factorises an approximate joint posterior into two densities over the covariance and model parameters: 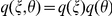. The moments (conditional mean and covariance) of the approximate posterior marginals, 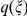 and 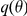, can then be updated iteratively under a fixed-form Laplace (i.e., Gaussian) approximation to the conditional densities; 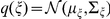
and 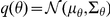. Under this assumption, the conditional covariances become analytic functions of the conditional means (see Protocol S1 in Text S1). This means the free energy becomes a function of the data and conditional means, which are optimised using a Gauss-Newton method:
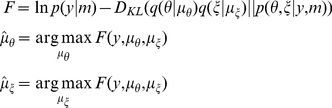
(9)where 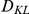 is the Kullback-Leibler divergence between the real and approximate posteriors. The conditional means are optimised iteratively until the change in free energy falls below 10^−2^. Note that Variational Laplace generalises previous variational schemes based upon expectation maximisation, which ignore condition uncertainty about the parameters optimised in the maximisation step: e.g., [69]. For dynamic models of the sort used in this paper, this approach has been evaluated in relation to Monte Carlo Markov chain (MCMC) techniques and has been found to be robust, providing accurate posterior estimates while being far superior to sampling schemes in terms of computational efficiency [70].

Given the empirical data, either from each animal separately (spectra from 10 to 35 Hz, comprising the auto-spectra of cortex, striatum, GPe, STN and their cross-spectra; Figure 4A), or the averaged cross-spectra over animals within either the control or lesioned group (the grand averaged data), we used this Variational Laplacian scheme to estimate both the log-evidence of the model (approximated by the free energy) and the posterior density over its parameters. The posterior or conditional densities of the model parameters provide variance estimates, which are used in Bayesian parameter averaging to weight individual parameter means. To quantify the 95% confidence intervals on these estimates the conditional covariance of the average is computed as the inverse of the sum of the inverse posterior covariance (i.e., precision) matrices. Note that while intrinsic parameters are optimised during the inversion, for simplicity, we focus on how Parkinsonian beta-activity is mediated by changes in the extrinsic effective connectivity, 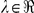, between nodes in the extended basal ganglia-cortical circuit.

### DCM Contribution Analysis

We used the MAP estimates above to analyse the response of the networks to small changes in circuit connectivity. Our goal was to see if particular connections (along the cortico-basal ganglia-thalamocortical pathways) contribute to beta frequencies more than others. This analysis assumes that the generative model of the spectra has been optimised and omits channel noise; i.e. using 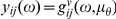 (Equations 3, 4 and 5 above) we computed the average effect of each connection on the beta peak centred at 17 Hz (summing from 16 to 18 Hz) using the derivative
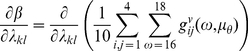
(10)


### Model Selection

As outlined above, the free energy is a lower bound on the model evidence [71] and is used for model selection, when testing a series of possible neural architectures using Bayes Factors (see Figure S2 in Text S1). The free energy can be rewritten in terms of the unknown parameters:
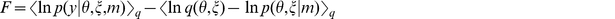
(11)This illustrates how maximisation of the free-energy also maximises the log-likelihood of the data expected under the (approximate) posterior. In classical model testing, goodness-of-fit comparisons are based on log-likelihood ratios. In this Bayesian setting, the more general ratio test uses the model evidence, which includes a penalty for any divergence between the prior and posterior densities; this divergence is complexity (the second term above). In short, the maximisation of free energy (which bounds log-evidence) ensures a maximum accuracy or data-fit, under complexity constraints. This free energy approximation to log-evidence has been shown to outperform other approximations such as the Bayesian information criterion [72] (BIC) and Akaike's information criterion [73] (AIC) in the context of DCM.

### Unit Circle Z-Transformation of a System's Impulse Response

A DCM can be seen as an input-state-output model of neuronal responses, where the white and pink noise inputs at each of sources (and four channels), renders the system a MIMO (*multi-input multi-output*) model. We can hence perform additional system identification, where all spectral properties of the BG-cortical circuits, as approximated by our model can be summarised. The system's transfer function can be constructed from the state-space (differential equation) formulation given above (Figure 3), using the Laplace transform, which has a polynomial form:
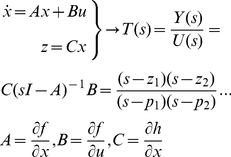
(12)Where, the matrices *A*, *B* and *C* depend on hidden parameters as in Equation 6. The transform uses the MAP estimates of the group DCMs. The Laplace transform uses the complex variable, 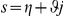, which, when evaluated at 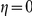, gives the frequency response. The ‘poles’ of the system correspond to the roots of the denominator (where an infinite output is observed). The system ‘zeros’ correspond to the roots of the transfer function's numerator and are where zero output will be observed. In our analysis, we employ a *z-*domain description, which samples the *s*-domain response to produce a discrete representation up to the Nyquist rate. The frequency response is immediately apparent in this representation because the power at each frequency, along the unit circle is given by the product of the distances from the point on the unit circle to each of the zeros, divided by the product of the distances from the point on the unit circle to each of the poles (illustrated in Figure 5B). A detailed description of these transforms can be found in [21]. This representation is provided for the *a posteriori* transfer functions of both the Control and Parkinsonian animals in Figure 5B. The z-transform introduces a nonlinear frequency warping under the bilinear approximation [74], such that continuous frequencies, *ω* are mapped in the sampled domain to 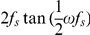 where 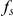 is the sampling frequency: For example, 20 Hz is mapped to 20.04 Hz and 60 Hz to 61.18 Hz. We use these graphs as qualitative references for further analysis (c.f. frequency spectra and gamma predictions, Figure 5B and see Figure S3 in Text S1).

## Supporting Information

Text S1 Additional model comparison, sensitivity analyses and robustness estimates. This explores a possible model space using Bayesian model comparison and presents additional sensitivity and robustness analyses that support our main conclusions.
(DOC)
