# Supplementary material for: Alterations in Brain Connectivity Underlying Beta Oscillations in Parkinsonism
Source: PLoS Comput Biol. 2011 Aug 11;7(8):e1002124. doi: 10.1371/journal.pcbi.1002124 (PMC3154892; doi:10.1371/journal.pcbi.1002124)
Supplement: Text S1 — Additional model comparison, sensitivity analyses and robustness estimates. This explores a possible model space using Bayesian model comparison and presents additional sensitivity and robustness analyses that support our main conclusions. (DOC) [file pcbi.1002124.s001.doc]

**Text S1**

**Alterations in brain connectivity underlying beta oscillations**

**in Parkinsonism**

Rosalyn J. Moran^1^, Nicolas Mallet^2^, Vladimir Litvak^1^, Raymond J. Dolan^1^, Peter J. Magill^2^, Karl J. Friston^1^, Peter Brown^3^.

^1^ Wellcome Trust Centre for Neuroimaging, Institute of Neurology, University College London, 12 Queen Square, London WC1N 3BG, UK.

^2^ Medical Research Council Anatomical Neuropharmacology Unit and Oxford Parkinson’s Disease Centre, University of Oxford, Oxford OX1 3TH, UK.

^3^ Department of Clinical Neurology, University of Oxford, Level 6, West Wing

John Radcliffe Hospital, OX3 9DU.

| **Supporting Information: Figure S1** | Overview of Findings |
| --- | --- |
| **Supporting Information: Table S1** | Table of model parameter’s values |
| **Supporting Information: Figure S2** | Model Comparison: Network Architecture |
| **Supporting Information: Figure S3** | Predicted Gamma |
| **Supporting Information: Figure S4** | Sensitivity Analysis: *Second 30s data set* |
| **Supporting Information: Figure S5** | Sensitivity Analysis: Parkinsonian *vs* Control network |
| **Supporting Information: Figure S6** | Sensitivity Analysis: 60 Hz oscillations |
| **Supporting Information: Figure S7** | Posterior Correlations |
| **Supporting Information: Figure S8** | Robustness Demonstration |
| **Supporting Information: Protocol S1** | EM Pseudocode |

**Figure S1**

|  | Change in PD | Predicted nature |
| --- | --- | --- |
| 1. | Increased strength (*c*) | Permissive of beta state^a^ |
| 2-4. | Increased potency ($d\beta/d\lambda_{A,B}$) | Promotes beta^b^ |
| 5. | Decreased strength (*c*) | Compensatory^b^ |

| **^a^ Therapeutic suppression might prevent plastic re-organisation & Parkinsonian state**  **^b^ Therapeutic suppression might alleviate established parkinsonism** |
| --- |

*Figure S1 Overview: Therapeutic Target, Schematic summary of network changes in PD*

Basic model as in Fig 1, but with changes in strength of effective connectivity (*c*) shown by altered arrow width and changes in the potency of such connectivity ($d\beta/d\lambda_{A,B}$*)* highlighted by red zig-zag arrows.

Table S1

| **Parameter** $\vartheta_{i}=\pi_{i}\exp(\Theta_{i})$ $\Theta_{i}=N(0,\sigma_{i})$ | **Interpretation** | **Prior**  **Mean: Variance:** $\pi_{i}$ $\sigma_{i}$ | |
| --- | --- | --- | --- |
| **Neuronal Sources** |  |  |  |
| $\rho_{1}$ $\rho_{2}$ | ***Sigmoid Shape parameter***  ***Sigmoid Position parameter*** | ***1***  ***2*** | ***1/8***  ***1/8*** |
| $H_{e/i}$ | ***Maximum Excitatory***  ***Maximum Inhibitory***  ***post-synaptic potentials (all regions)*** | ***8***  ***32*** | ***1/8***  ***1/8*** |
| $\kappa_{e/i}$ | ***Excitatory***  ***Inhibitory***  ***Rate Constants*** | ***4***  ***16*** | ***1/8***  ***1/8*** |
| $\gamma_{1,2,3,4,5}$ | ***Intrinsic Cortical Connections***  ***1. Pyramidal cells to stellate cells***  ***2. Stellate cells to Pyramidal cells***  ***3. Pyramidal cells to Inhibitory interneurons***  ***4. Inhibitory interneurons to Pyramidal cells***  ***5. Reciprocal Inhibitory connections*** | ***128***  ***128***  ***64***  ***64***  ***4*** | ***1***  ***1***  ***1***  ***1***  ***1*** |
| $\lambda_{A,B}$ | ***Excitatory Extrinsic Connections*** | ***32*** | ***1/2*** |
| $\lambda_{A,B}$ | ***Inhibitory Extrinsic Connections*** | ***16*** | ***1/2*** |
| $D_{ext}$ | ***Extrinsic Delay*** | ***4*** | ***1/8*** |
| $d_{\mathrm{int}}$ | ***Intrinsic Delay Between Cortical Layers*** | ***2*** | ***1/32*** |
| $\alpha_{u}$ $\beta_{u}$ | ***Exogenous White Input***  ***Exogenous Pink Input*** | ***1***  ***0.4*** | ***1/2***    ***1/2*** |
| **Observation Model** |  |  |  |
| $\alpha_{ctx}$ $\beta_{ctx}$ | ***White Noise at cortical channel***  ***Pink Noise at cortical channel*** | ***1***  ***1*** | ***1/16***    ***1/16*** |
| $\alpha_{BG}$ $\beta_{BG}$ | ***White Noise at BG channel***  ***Pink Noise at BG channel*** | ***1***  ***1*** | ***1/16***    ***1/16*** |
| $\alpha_{comm}$ $\beta_{comm}$ | ***White Noise common to all BG channels***  ***Pink Noise common to all BG channels*** | ***1***  ***1*** | ***1/16***    ***1/16*** |

*Table S1 Priors*

Priors for model parameters including the observation model and neuronal sources. To ensure positivity, we estimate the log of these parameters under Gaussian priors on their log-scaling. This is equivalent to adopting a log-normal prior on the constants *per se*, where the parameter is $\vartheta_{i}=\pi_{i}\exp(\Theta_{i})$ with $\Theta_{i}=N(0,\sigma_{i}^{2})$, $\pi_{i}$ is the prior expectation and $\sigma_{i}^{2}$ is its log-normal dispersion (David et al., 2005).

**Figure S2**

*Figure S2 Model Comparison*

Here we show the results of a fixed effects model comparison (Stephan et al., 2009) for Control and Parkinsonian animals. Bayesian model comparison, using the approximate model evidence allows us to compare competing hypotheses about the neural architecture generating the data. For each animal, we inverted or fit the data using three models. This inversion estimates a lower bound on the log model evidence, known as the free energy, it accounts for both the accuracy of fit and complexity of each model. Model 1 comprised the “standard” Basal-Ganglia-Thalamocortical re-entrant pathways described in the main text. Model 2 comprised this “standard” architecture and a new connection from GPe to EPN. Model 3 comprised the “standard” architecture and a new connection from GPe to striatum. Here we show there is very strong evidence in favour of Model 1, with a Group Bayes Factor (*GBF_1,2_*) > 150 (i.e. >99% probability, Penny et al., 2004) for Model 1 relative to the next best performing model (Model 2). For the Control animals the *log* Bayes factor (illustrated) *logGBF_1,2_* = 167.02 and for the Parkinsonian animals *logGBF_1,2_* = 20.10.

**Figure S3**

*Figure S3 Predicted Gamma*

We examined the frequency structure produced by model inversion using pole zero plots (Fig. 5B). The frequency structure was produced by the modulation transfer function, found using only data from 10 – 35 Hz. In the case of the control animals, the model predicted increased gamma output. We hence examined the frequency content of the original data from each animal over 40 – 80 Hz and found the predicted gamma peak in control animals (red) in Basal Ganglia nuclei.

**Figure S4**

*Figure S4 Sensitivity Analysis: Second 30s data set*

Here, we show the results of the second sensitivity analysis, having obtained *a posteriori* connectivity estimates using a new 30s data epoch from each animal. The results are similar to those of Figure 6A and show that connections from Striatum to GPe and from GPe to STN significantly increase beta activity in the Parkinsonian network (***p* <0.005; **p*< 0.05 *Bonferroni corrected for multiple comparisons*).

**Figure S5**


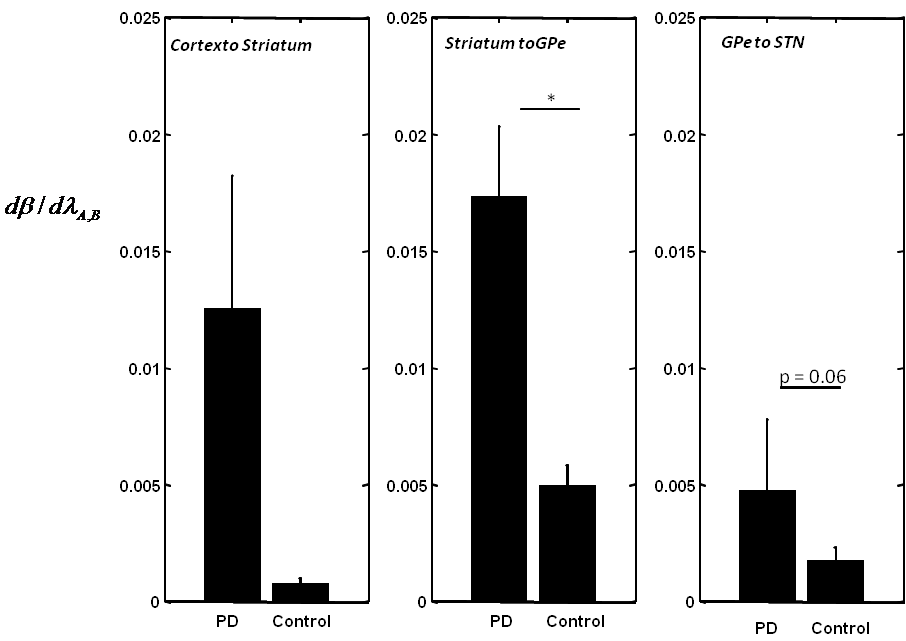


*Figure S5 Sensitivity Analysis of connections in the indirect pathway: Parkinsonian vs. Control network*

Comparison of $\partial\beta/\partial\lambda_{A,B}$for healthy and Parkinsonian networks along indirect pathway connections, cortex to striatum, striatum to GPe and GPe to STN. Although the connections did not show significant strength differences, (main text Figures 4B and 5A), perturbations in these connections increased beta activity when embedded in the Parkinsonian network (**p<*0.01 *Bonferroni corrected for multiple comparisons*).

**Figure S6**


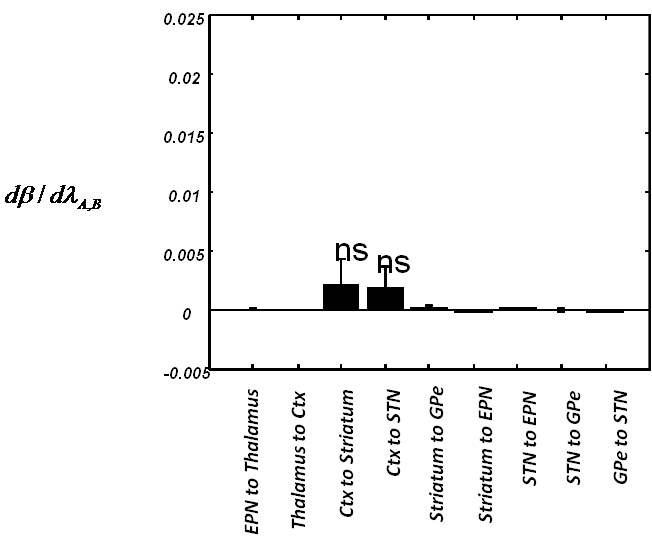


*Figure S6 Sensitivity Analysis: 60 Hz oscillations*

Here we show the results of a sensitivity analysis, where we measure the sensitivity of a non beta frequency (60 Hz gamma) to changes in connection strength for the Parkinsonian animals. The results should be compared to Fig 6A. We find no significant sensitivity across animals in any connection.

**Figure S7**

*Figure S7 Posterior Correlations and Parameter Identifiability*

Here, we show the posterior correlation matrices derived from the posterior covariance of the averaged Control data DCM and averaged Parkinsonian data DCM. High correlations in posterior estimates indicate redundancy in the network, where two parameters may contribute equivalently to the observed response. The colorbar is applicable to all images. *Top.* Control DCM correlations have high values (0.91) for parameter set *γ*; the intrinsic cortical connections rendering these parameters unidentifiable. However, for our parameters of interest; the extrinsic connectivity parameters λ, the average correlation of parameters is 0.12 ± 0.19 (mean ± std), with a maximum (absolute) correlation between parameters *λ_3,2_* (parameter 5 striatum to GPe) and *λ_3,4_* (parameter 8 STN to GPe) of 0.66 (inset). *Bottom.* Similarly for the Parkinsonian DCM, the maximum correlation is observed for the intrinsic cortical connectivities *γ* (-0.82). While for the parameter set λ, correlations are in general low (0.0649 ± 0.1396), with maximum (absolute) correlation at (0.45) for parameters *λ_4,1_* (parameter 4 cortex to STN) and *λ_3,2_* (parameter 5 striatum to GPe). Furthermore, when examining the key parameters λ in the individual DCMs for the Control and Parkinsonian animals, we found maximum posterior correlations among parameter pairs that varied from data set to data set; with three different pairs exhibiting maximal correlations among the Control animals and six different pairs showing maximal correlation in the Parkinsonian models. While ideally parameter correlations would be zero, an average of ~0.1 suffices for making meaningful conclusions. Overall these data highlight the identifiability of the extrinsic connectivity parameters.

**Figure S8**

*Figure S8 Robustness Demonstration*

MAP estimates plotted with 95% Bayesian credible intervals for a new model where connections from STN to GPe were omitted. The DCM was fit to the grand averaged Control (white) and PD (black) data separately. We see that although the parameters are optimised to different posterior values (compared to the standard model; Figure 5A), the same connection remains significantly different (Cortex to STN) between groups (>95% probability).

**Protocol S1 EM Pseudocode**

Following Friston et al., (2006; 2002) our E-step employs a local linear approximation of Equation 3 (main text) about the current conditional expectation $q(\theta)\sim N(\mu,\Pi^{-1})$

$$\begin{matrix} y-h(\mu)\approx\Im\Delta\theta+\varepsilon\\ \\ \Delta\theta=\theta-\mu\\ \Im=\frac{\partial h(\mu)}{\partial\theta} \end{matrix}$$

This local linear approximation allows one to perform a gradient ascent on the free energy to optimise the posterior moments, given the priors $\Theta=N(0,\sigma)$ (see Table S1 in Text S1). The M-step performs an ascent on the free energy to update the hyperparameter. This is repeated until convergence where the objective function *F* changes by less than 10^-2^.

**E-step**

$\begin{matrix} \begin{matrix} \overline{y}=\left[\begin{matrix} y-h(\mu) \\ -\mu\end{matrix}\right] & \overline{\Im}=\left[\begin{matrix} \Im\\ I \end{matrix}\right] & \overline{C}=\left[\begin{matrix} \xi V & 0 \\ 0 & \sigma\end{matrix}\right] \end{matrix} \\ \\ \Pi^{-1}=(\overline{\Im}^{T}\overline{C}^{-1}\overline{\Im})^{-1} \\ \Delta\mu=\Pi^{-1}(\overline{\Im}^{T}\overline{C}^{-1}\overline{y}) \end{matrix}$

**M-step**

**References**

Penny WD, Stephan KE, Mechelli A, Friston KJ, (2004). Comparing dynamic causal models. Comparing Dynamic Causal models. Neuroimage, 22, 1157-1172.

Stephan KE, Penny WD, Daunizeau J, Moran RJ, Friston KJ, (2009) Bayesian model selection for group studies. Neuroimage, 46, 1004-1017

David O, Kiebel SJ, Harrison LM, Mattout J, Kilner JM, Friston KJ. (2006) Dynamic causal modeling of evoked responses in EEG and MEG. Neuroimage, 30, 1255-1272.

Friston KJ, Mattout J, Trujilo-Barreto N, Ashburner J, Penny W. (2006) Variational free energy and the Laplace approximation. Neuroimage 34, 220-234.

Friston KJ, Glaser DE, Henson RNA, Kiebel S, Phillips C, Ashburner J. (2002) Classical and Bayesian Inference in Neuroimaging: Applications. Neuroimage, 16, 484 -512.
